# Updates and Current Knowledge on the Common Forms of Gastroenteritis: A Review

**DOI:** 10.3390/jcm14103465

**Published:** 2025-05-15

**Authors:** Pranav Patel, Hareesha Rishab Bharadwaj, Omar Al Ta’ani, Shahryar Khan, Saqr Alsakarneh, Sheza Malik, Umar Hayat, Manesh Kumar Gangwani, Hassam Ali, Dushyant Singh Dahiya

**Affiliations:** 1 Department of Hospital Medicine, Prisma Health Greenville Memorial Hospital, Greenville, SC 29605, USA; 2 Faculty of Biology Medicine and Health, University of Manchester, Manchester M13 9PL, UK; 3 Department of Internal Medicine, Allegheny Health Network, Pittsburgh, PA 15202, USA; 4 Department of Internal Medicine, University of Kansas Health System, Kansas City, KS 66160, USA; 5 Department of Internal Medicine, University of Missouri, Kansas City, MO 641110, USA; 6 Department of Internal Medicine, Rochester General Hospital, Rochester, NY 14621, USA; 7 Department of Internal Medicine, Geisinger Wyoming Valley Medical Center, Wilkes-Barre, PA 18711, USA; 8 Department of Gastroenterology and Hepatology, University of Arkansas for Medical Sciences, Little Rock, AR 72205, USA; 9 Division of Gastroenterology, Hepatology & Nutrition, Brody School of Medicine, Greenville, NC 27834, USA; 10 Division of Gastroenterology, Hepatology & Motility, University of Kansas School of Medicine, Kansas City, KS 67214, USA

**Keywords:** acute gastroenteritis, viral gastroenteritis illnesses, bacterial gastroenteritis, *Shigella*, norovirus, sapovirus

## Abstract

**Background/Objective:** Acute gastroenteritis is a major cause of diarrheal illnesses throughout the United States. The purpose of this article is to review the current knowledge in diagnostic and therapeutic aspects. **Methods:** A comprehensive literature review was conducted using PubMed and Google Scholar, focusing on articles published within the last ten years. **Results:** There are multiple etiologies of gastroenteritis that affect the general population. Out of the many causes, norovirus continues to be a leading cause of acute diarrheal illness worldwide. Rotavirus was also a common form of diarrhea worldwide, but the development of routine vaccination has largely reduced its incidence. Bacterial gastroenteritis continues to be a significant burden on healthcare facilities worldwide. Supportive care remains the cornerstone of treatment, while using antibiotics remains crucial in severe bacterial forms of gastroenteritis. **Conclusions:** Acute gastroenteritis remains a significant global health concern requiring a multifaceted approach for effective management. Enhanced diagnostic techniques, vaccine development, and robust public health measures are essential in controlling the spread of gastroenteritis.

## 1. Introduction

Acute diarrheal illnesses cause millions of infections in the United States annually [1]. Many of these illnesses are viral, however, there are opportunistic infections that affect many patients nationally. Although most gastroenteritis infections are self-limited, pathogen evolution and antibiotic resistance pose treatment challenges to many clinicians [1]. Thus, this article will seek to highlight the most common etiologies for acute gastroenteritis and discuss the disease course, epidemiologic data, risk factors, and treatment modalities available. We will also discuss areas of future research in terms of diagnosis and treatment.

## 2. Materials and Methods

A comprehensive literature search was conducted on 6 January 2025, using PubMed (https://pubmed.ncbi.nlm.nih.gov/) (accessed on 6 January 2025) and Google Scholar databases (https://scholar.google.com/) (accessed on 7 January 2025). Search terms included: “Acute gastroenteritis”, “viral GI illnesses”, “treatment outcomes”, and “epidemiology of gastroenteritis illnesses”. The search was limited to articles published in the last 10 years (2015–2025), written in English, and involving human subjects.

A total of 147 articles were initially retrieved. After title and abstract screening, 87 full-text articles were assessed for eligibility. Ultimately, 62 studies were included in this review based on the following criteria. Studies that focused on epidemiology, pathogenesis, clinical presentation, diagnostics, treatment, or prevention of viral and bacterial causes of gastroenteritis. Studies providing updated clinical guidelines or recent public health data. Non-English language publications and publications older than 10 years were excluded.

The selected literature was analyzed thematically to synthesize current evidence and trends in the diagnosis, treatment, and prevention of gastroenteritis across various pathogens.

## 3. Results

### 3.1. Epidemiology

The most frequent cause of diarrheal illnesses worldwide is acute viral gastroenteritis [2]. Out of the many viral etiologies, norovirus remains the most common cause [2].

Although the exact timing is unclear, the rise of norovirus as the dominant form of gastroenteritis occurred after rotavirus vaccines were introduced. Before the development of routine vaccination, the most common cause of viral diarrheal illness worldwide was rotavirus in the pediatric population. However, since the implementation of vaccination in 2006, the number of cases seen in the United States has declined 50–90% per year [3]. The other common pathogens causing viral gastroenteritis are enteric adenovirus and astrovirus [4]. Other non-viral causes include bacteria such as non-typhoidal *Salmonella*, *Campylobacter jejuni*, *Shigella* spp., *Yersinia*, *Clostridium difficile,* and *Escherichia coli,* and parasites like Giardia and Cryptosporidium [5].

### 3.2. Norovirus

#### 3.2.1. Epidemiology

Norovirus (NoV) is the leading cause of gastroenteritis across all age groups worldwide [6]. Ever since its discovery, NoV has been recognized as a primary source of gastroenteritis, and it has contributed to approximately 200,000 deaths annually (predominantly in underdeveloped countries). Its ability to mutate and adapt to its environment within and outside of a host makes it immensely virulent and puts immense pressure on our healthcare services [6]. In addition, the virus thrives in cooler environments, making it more common during the winter months [7].

#### 3.2.2. Transmission

Norovirus can be spread easily due to an infected individual’s ability to shed billions of viral particles via feces or oral mucosa, which can in turn contaminate food, water, and surfaces [8]. It is highly contagious due to its low infectious dose, being as little as 18 viral particles [8]. The two age groups that face the largest risk of acquiring NoV are children below the age of 5 and the elderly above the age of 85, with the elderly group being at a much higher risk [9,10,11]. As a result, the most common sites of outbreaks include long-term care facilities and childcare centers, in addition to restaurants and cruise ships [8]. It is thought that the elderly and children are more prone to these infections due to their immunosenescence and higher risk for developing dehydration [12].

Of note, norovirus is subdivided into 10 genogroups and 49 genotypes based upon sequence homology. Genogroups GI, GII, and GIV include human pathogens, and frequent recombination between strains contributes to rapid changes in genetic diversity, although illnesses caused by each genogroup are clinically indistinguishable. The most common cause of human norovirus infection is GII (GII.4 followed by GII.7), followed by GI and GIV. GII.4 viruses are predominant and have the most severe outcomes when compared to other norovirus genotypes, including higher hospitalization and death rates [10,13,14,15].

#### 3.2.3. Diagnosis, Clinical Presentation, and Treatment

Once acquired, the life cycle and presentation of the virus varies. It is currently estimated that norovirus’s incubation period is estimated to be between 10–51 h [16]. Once active, patients are split into asymptomatic and symptomatic types. Although the exact mechanism for norovirus-induced vomiting and diarrhea remains unclear, the virus begins by causing reversible damage to the microvilli in the jejunum, with apparent sparing of the stomach and rectum. Diarrhea symptoms are induced by D-xylose and fat malabsorption, with enzymatic dysfunction observed in the brush border [17]. The virus then affects gastric motility and causes delayed gastric emptying, thus leading to vomiting symptoms.

Typical clinical features of norovirus include nausea/vomiting (non-bloody, non-bilious), watery diarrhea, and abdominal pain [15]. Additionally, generalized myalgias, malaise, and headache are prominent [15]. With the above clinical features, a norovirus infection can be suspected in patients who reside in middle- or high-income countries where rotavirus vaccines are routinely used [15]. A confirmed diagnosis can be obtained by using a multi-pathogen molecular test for gastrointestinal pathogens [15]. The benefits of obtaining a confirmed diagnosis can aid public health laboratories in tracking outbreaks and aid in prevention.

Once confirmed, the treatment consists of supportive measures, including fluid repletion and adequate nutrition. The key to reducing disease severity is to maintain adequate hydration. Additionally, antiemetics and antimotility agents can be used intermittently to treat excessive vomiting and diarrhea symptoms, respectively [15]. Due to its viral etiology, antibiotics do not have a role in treatment, and thus, prevention plays a key role in avoiding disease outbreaks. Due to its virulent nature and transmissibility, the best prevention method is adequate hand hygiene. Infections occurring in healthcare and long-term care facilities involve establishing contact/barrier (i.e., gloves) precautions in addition to maintaining adequate hand hygiene [5]. Infections identified secondary to food outbreaks require significant public health measures to be directed at the source of the outbreak [5].

In most cases, norovirus-induced diarrheal illness has an excellent prognosis; however, complications do arise in certain susceptible populations. Those include older frail adults who are susceptible to dehydration and complications such as syncope and hypotension. These patients require closer follow-up and a lower threshold for hospitalization [5]. Furthermore, patients are prone to develop post-infectious irritable bowel syndrome (IBS) and post-infectious functional dyspepsia [5]. Risk factors for developing post-infectious IBS included female sex, anxiety, duration of diarrhea greater than 3 weeks, and hospitalization [5]. Although functional dyspepsia is well described after infectious gastroenteritis, the etiology of this is not well understood. Notably, patients who acquire norovirus-induced gastroenteritis are at the highest risk of developing functional dyspepsia when compared to other pathogens [18].

### 3.3. Rotavirus

#### 3.3.1. Epidemiology

Rotavirus is the most common cause of severe gastroenteritis in infants and young children worldwide. Prior to the introduction of rotavirus vaccines in the United States, the virus caused an estimated 2.1 to 3.2 million diarrheal illnesses each year, with 55,000–70,000 of those cases requiring hospitalization annually [19]. Since the introduction of the vaccine, 280,000 clinic visits and 62,000 emergency department visits were averted [20]. Although the incidence of cases in the US decreased, the worldwide incidence of Rotavirus is estimated to cause more than 125 million cases annually [21].

#### 3.3.2. Transmission

Rotavirus is transmitted similarly to norovirus, where person-to-person transmission is mainly via the fecal–oral route. Contaminated surfaces (including door-handles, water-taps), airborne droplets, and contaminated food and water sources are other common routes of transmission [22]. Children between the ages of 4 to 24 months, particularly those in daycare settings, are at increased risk for rotavirus infections [21]. By the age of 3 to 5, almost every child worldwide has been infected with rotavirus. Although adults are also prone to infection, most are often asymptomatic or less severely infected. Notably, rotavirus has six distinct groups (A through F); however, Group A rotavirus represents greater than 95% of isolated strains in humans worldwide. In the Group A subgroup, the G1 and G12 strains account for the majority of infections [20].

#### 3.3.3. Diagnosis, Clinical Presentation, and Treatment

The virus has a short incubation period of less than 48 hrs, and once active, patients present with general viral gastroenteritis symptoms such as watery diarrhea, fever, nausea, and vomiting [20]. The severity of infection is usually higher in children greater than 3 months, where, comparatively, infants younger than 3 months have relatively low rates of rotavirus, likely due to passive maternal antibodies or due to breastfeeding [20].

Although it is not possible to diagnose rotavirus by clinical presentation alone, there are confirmatory tests available. The most commonly used test is ELISA (enzyme-linked immunosorbent assay) with a sensitivity and specificity of 88% and 94%, respectively, and ICT (immunochromatography) with a sensitivity and specificity of 75% and 95%, respectively [22,23]. Most rotavirus infections do not require a confirmatory diagnosis, but clinical management can be guided by presenting symptoms. Children usually present with symptoms of nausea, vomiting, non-bloody diarrhea, and fever, with the average duration of symptoms being 8 days [24]. Adults who are affected by rotavirus usually obtain the infection from household members of affected children and have a much milder disease course [24]. Lab findings are usually normal; however, dehydrated patients can present with an elevated blood urea nitrogen and metabolic acidosis [24]. Outbreaks of rotavirus are common in childcare centers or settings with many young children [20]. Rotavirus is primarily transmitted through the fecal–oral route, but due to stability in the environment, transmission can also occur via ingestion of contaminated water or food and contact with contaminated surfaces or objects [20]. Notably, only a small inoculum is needed for transmission, where no more than 100 focus-forming units (FFU) are required for infection [24]. Although rotavirus infection can occur year-round, peak infections are noted during the cooler winter months [24].

Treatment for rotavirus-induced gastroenteritis usually focuses on supportive measures, which include adequate fluid repletion and nutrition. In moderate cases without signs of volume depletion, patients can maintain adequate volume with sports drinks and broths. In more severe cases with signs of hypovolemia, oral rehydration solutions (i.e., Pedialyte, Ceralyte) may assist in establishing adequate hydration [24]. Fortunately, oral rehydration solutions are available over the counter, which makes alternate hydration options accessible for recovering patients. Symptoms of nausea, vomiting, and diarrhea can be treated with antiemetics and antidiarrheal agents, respectively. Rotavirus infections can be prevented by maintaining adequate hand hygiene, contact precautions, and vaccination. There are two live, attenuated oral rotavirus vaccines available in the United States, Rotateq and Rotarix, and both have been reported to have similar efficacy and safety [25]. Importantly, the above vaccines are only available for infants, where all doses should be administered before 8 months of age. The Rotavac vaccine is licensed for use in India and resource-limited countries, and in addition, Rotasiil is also available worldwide for administration [25]. It is important to note that the Lanzhou lamb rotavirus vaccine is licensed in China, which is reported to have 57% effectiveness in reducing rotavirus gastroenteritis and 70% effectiveness in reducing severe rotavirus gastroenteritis [25]. Additionally, Rotavin-M1 is offered in Vietnam, having been licensed for administration in 2012 [25]. Since the development and introduction of these vaccines, the incidence of rotavirus infections in the United States has drastically improved. Thus, continuing to encourage vaccine administration to newborn parents will be key in preventing future infections.

### 3.4. Astrovirus, Sapovirus, and Enteric Adenovirus

#### 3.4.1. Epidemiology

Astrovirus, sapovirus, and adenovirus collectively account for 2–9% of viral gastroenteritis cases worldwide [2]. Although their incidence rates compared to norovirus and rotavirus are low, these viruses still cause clinically significant diarrheal illnesses worldwide.

#### 3.4.2. Transmission, Clinical Presentation, and Treatment of Astrovirus

Astrovirus commonly tends to affect young children less than 2 years of age, with low incidence rates in older children and adults. The rate of incidence remains the same in developed and underdeveloped countries, with astrovirus diarrhea cases peaking during the winter months [26]. Outbreaks have been reported in schools, day care centers, hospitals, nursing homes, and households [26]. The virus is transmitted via the fecal–oral route, contaminated water, food, and surfaces [27]. Once acquired, the incubation period is 3–4 days, although it has been noted that children may shed virus 1–2 days prior to illness and 4–5 days following the illness. The clinical course is characterized by 2–5 days of watery diarrhea, which is often accompanied by nausea/vomiting [27]. The illness is generally self-limited, and symptoms are usually less severe compared to rotavirus. Most patients improve with oral rehydration and symptomatically treating nausea and vomiting symptoms with antiemetics. The key to prevention remains similar to other viral diarrheal illnesses, which includes appropriate handwashing, contact precautions, and ensuring contaminated surfaces are cleaned adequately [26].

##### Transmission, Clinical Presentation, and Treatment of Sapovirus

Sapovirus, a genus of the *Calciviridae* family like norovirus, is increasingly recognized as an important cause of childhood diarrhea. Although we have a general understanding of this family of viruses, there are challenges that exist in our ability to grow sapovirus in cell culture, which, in return, has hindered diagnosis and indirectly affected how we measure clinical incidence. Presently, the prevalence of sapovirus varies between 1–17% of diarrheal episodes worldwide, where it mostly affects children under the age of 5, and older adults > 60 years of age. Sapovirus is usually diagnosed with reverse transcription (RT)—PCR methods, but testing is usually not performed due to cost and lack of access around the world [28]. The most common genotype causing clinically significant infections is the GI.1 variant, followed by the GI.2 and GII.1 variants [28]. The virus is spread through wastewater intrusion of drinking water sources, contaminated food, or via individuals who live in the same household [28]. Among foodborne transmission, shellfish is a well-recognized source of infection [28]. Compared to other gastroenteritis infections, sapovirus infections can occur throughout the year. The most common clinical symptoms include diarrhea, nausea, vomiting, and abdominal pain. The virus is self-limited and usually resolves within one week [28]. Currently, there are no vaccines to prevent future sapovirus infections, and treatment involves supportive measures, including oral rehydration and symptomatic treatment of nausea/vomiting with antiemetics. Patients who develop severe dehydration symptoms should pursue rapid intravenous rehydration. Although little is known about the environmental decontamination of sapoviruses, similar measures used to control norovirus can be pursued. This is likely to be successful as both viruses belong to the same family. Prevention revolves around regular hygiene, including frequent handwashing, proper disposal of fecal or soiled materials, limited contact with infected individuals, and disinfection of contaminated surfaces with chlorine-containing cleaning agents [28].

##### Transmission, Clinical Presentation, and Treatment of Adenovirus

Adenovirus is a common cause of multiple infections worldwide. It most commonly causes upper respiratory syndromes such as pharyngitis or coryza, but it has also been implicated in causing gastrointestinal, ophthalmologic, genitourinary, and neurologic diseases as well [29]. In terms of gastroenteritis, adenovirus accounts for 2–6% of infections worldwide [30]. Due to the fact that adenovirus can cause multiple different infections, certain genotypes of the virus are associated with particular infections. Genotypes A (serotype 31) and F (serotype 40, 41) are responsible for causing gastroenteritis [29]. The tests used to confirm infection are adenovirus-specific polymerase chain reaction (PCR) or enzyme-linked immunosorbent assay (ELISA) on a stool specimen, as enteric adenoviruses do not routinely grow on routine tissue culture [29]. The virus is spread via close contact with infected persons, contact with contaminated surfaces, and handling an infected person’s stool. Compared to other diarrheal illnesses, adenovirus also does not have a seasonality and can occur year-round [29]. Although the prevalence of adenovirus is high in children from infancy to school age, children of any age can be affected, including young adults living in close quarters [31]. Symptoms usually involve diarrhea, fever, nausea, and vomiting, with most symptoms resolving within 1 to 2 weeks of onset. Fortunately, most infections are self-limited, and treatment involves supportive care with adequate fluid hydration and symptomatically treating fever, nausea, and vomiting symptoms with antipyretics and anti-nausea medications, respectively. Prevention is key when trying to control disease spread, and similar strategies, such as adequate hand hygiene and disinfecting contaminated surfaces, should be employed. Although a live oral vaccine is available, this was only developed to target serotypes 4 and 7 to prevent acute respiratory illnesses. This vaccine is only given to military recruits due to a high probability of adenoviral upper respiratory infections in close quarters [31].

Table 1 summarizes the viruses discussed in this review, including their incidence, seasonality, treatment, and prevention:

### 3.5. Bacterial Etiologies of Gastroenteritis

There is a wide array of pathogens implicated in causing acute bacterial gastroenteritis. Compared to viral etiologies, bacterial causes are responsible for severe cases of diarrhea. Among these bacterial causes, non-typhoidal *Salmonella* and *Campylobacter* are the most common causes in the United States, followed by *Shigella* and *Escherichia coli* [32].

#### 3.5.1. Non-Typhoidal *Salmonella*

##### Epidemiology

Non-typhoidal *Salmonella* remains a significant public health problem in the United States and around the world. There are many serotypes of *Salmonella*, but the most common strains responsible for acute gastroenteritis are *Salmonella enterica* serotype Enteritidis, *S. typhimurium, S. newport, S. heidelberg, and S. javiana*, where the Enteritidis and Typhimurium variants are the most common [33,34]. The annual burden of non-typhoidal *Salmonella* in the United States is about 1.35 million cases annually, with 420 reported deaths [35].

##### Transmission

The pathogen remains virulent due to its ability to survive in a wide range of conditions, where it can grow in temperatures ranging from 8 °C to 45 °C and in environments with a pH ranging from 4.0 to 9.5 [34]. Animals are considered to be the primary disease vectors when transmitting the infection to humans, where the most common sources of infections are beef, poultry, dairy, and eggs [34,36]. Although the infectious dose varies among different *Salmonella* strains, a large inoculum is thought to be needed in order to overcome stomach acidity and to compete with normal intestinal flora [36]. Once infected, the virus subverts our innate immunity by bypassing the gastric acid barrier and invading the intestinal epithelial cells, initiating a chain reaction leading to infection [36]. The incubation period depends on the host, but it is usually observed to be from 6 to 72 h [36].

##### Diagnosis

Of the several lab studies available for confirmation, polymerase chain reaction (PCR) allows for rapid identification and diagnosis via testing of primary stool specimens [34]. Approximately 90% of isolates are obtained from routine stool cultures. Other ways to obtain isolates are from blood cultures, abscesses, cerebrospinal fluid, and urine [37]. Once active, patients present with loose stools, which are usually bloodless, in addition to fever, abdominal cramping, chills, headaches, and myalgias [36]. The populations most prone to acquiring *Salmonella* infections are children younger than 5 years, with a peak among those younger than 1 year. Infants and people older than 60 are the most susceptible to more severe infections [36]. Fortunately, most non-typhoidal *Salmonella* infections are self-limited, and patients recover from the illness within 4 to 7 days of disease onset [34].

Although most *Salmonella* cases are self-limited, it is important to note that immunocompromised hosts are at a much higher risk of invasive *Salmonellosis.* Infants and people older than 60 years are the most vulnerable to developing invasive infections. The most critical forms can involve bacteremia or focal invasive infections such as osteomyelitis, meningitis, endovascular infections, or septic arthritis. Notably, the rates of invasive (extra-intestinal disease) infections increase in patients with HIV, hemoglobinopathies (i.e., Sickle Cell), atherosclerosis, or malignant neoplasms [37].

Complications

As mentioned above, extra-intestinal spread of *Salmonella* can cause severe infections.

- **Endovascular infection:** Although an uncommon manifestation, the organism has a tendency to attach at sites with atherosclerosis of large vessels [38]. The abdominal aorta is the most frequent site of vascular infection, followed by the thoracic aorta. Clinical manifestations include subacute fever, abdominal pain, and back pain [38].
- **Endocarditis:** This is a rare complication and usually involves prosthetic valves or patients with underlying valvular disease [38].
- **Septic arthritis:** The disease can affect native or prosthetic joints. Due to its nature of involvement, it can often be difficult to eradicate [38].
- **Osteomyelitis:** *Salmonella* osteomyelitis affects children, and those with Sickle Cell Anemia are the most susceptible. Notably, it typically involves the epiphyses of long bones [39].
- **Meningitis:** A rare complication affecting neonates and children < 12 months of age. Infected patients typically present with fever and lethargy. Complications include hydrocephalus and ventriculitis [38]. In populations with high HIV prevalence, non-typhoidal *Salmonella* has also been described in adults [38].

##### Treatment

The management of invasive non-typhoidal *Salmonella* differs from its non-invasive form. While the non-invasive form is self-limited, patients affected by invasive *Salmonella* are given a prolonged course of antibiotics based on the infection site. Patients with endovascular infections, endocarditis, septic arthritis, or central nervous system involvement receive at least six to eight weeks of antimicrobial therapy [38]. Of note, although antibiotics are the mainstay of therapy, certain invasive infections (endovascular, septic arthritis) should be considered for surgical debridement if applicable. The involvement of a skilled surgeon will be key in management. Treatment revolves around antibiotics that provide coverage against Gram-negative bacteria, including third-generation cephalosporins, including Ceftriaxone, Cefotaxime, and fluoroquinolones such as Ciprofloxacin and Levofloxacin [38]. Other antibiotics that can be used are Trimethoprim-Sulfamethoxazole or Ampicillin [38]. It is important to note that antibiotics should be tailored based on culture data and susceptibility results. Comparatively, immunocompetent patients with *Salmonella* bacteremia complicating acute gastroenteritis can be given a 10–14 day course of antibiotics [38]. It is important to note that immunocompromised hosts have the highest risk of relapse and thus should be given a prolonged course of treatment.

Prevention plays a key role in mitigating spread. In order to reduce disease burden, concerted efforts should be taken to maintain proper hand hygiene, sanitation, and avoidance of insufficiently cooked food. Non-typhoidal *Salmonella* infections are a source of significant public health concern, and despite aggressive efforts, they continue to contribute to foodborne illness worldwide. In order to continue decreasing disease incidence, a multi-faceted approach will be necessary. This includes stringent food safety regulations, public education on safe food handling, and appropriate antimicrobial stewardship to prevent the emergence of resistant strains.

#### 3.5.2. *Campylobacter jejuni*

##### Epidemiology

*Campylobacter* is the second leading cause of clinically significant foodborne infections in the United States. According to the Centers for Disease Control and Prevention’s active surveillance program, Food Net, *Campylobacter* had an incidence rate of 11.8% per 100,000 persons [32]. In 2023, the incidence of *Campylobacter* was 18.2 infections per 100,000 persons [40]. Although comparatively higher than 2016, the rate of incidence had dropped compared to 2022, when the incidence was 19.2 per 100,000 persons. Interestingly, there is a variation in incidence within the United States. The western states have a higher incidence of infection compared to states in the eastern US [41]. Although the disease is prevalent, mortality due to *Campylobacter* remains low. In the United States, there are an estimated 50–150 deaths annually that are attributed to *Campylobacter.*

##### Transmission

The major species of *Campylobacter* are *C. jejuni*, *C. Coli*, *C. fetus*, *and C. Lari* [42]. The pathogen is prevalent in animals, including cattle, poultry, pigs, ostriches, sheep, and pets such as cats and dogs [43]. Transmission to humans occurs via consumption of contaminated food, water, and person-to-person contact. Most human infections result from consuming contaminated or improperly cooked food [42]. Children younger than 4 and individuals between 15 to 44 are commonly affected [42]. Notably, the disease incidence rises sharply in the summer with a steady decline in the winter months [41]. Once acquired, most *Campylobacter* infection begins to appear after an incubation period of 1 week. The pathogen invades the jejunum, the ileum, and often the colon and rectum by invading the intestinal epithelial cells [41]. Once infected, the flagella play a key role in chemotaxis and adherence to the epithelial cell lining. This adherence promotes gut colonization, and following attachment, several bacterial surface proteins (PEB1, CadF) help the organisms colonize and then invade the intestinal epithelial cells [41].

##### Diagnosis, Clinical Presentation, and Treatment

Acute *Campylobacter* infections can be confirmed with stool culture, enzyme immunoassay (EIA), or polymerase chain reaction (PCR). Out of the various testing methods that are available, the use of reverse transcription-PCR is rapidly expanding [41,42]. Once infected, patients can present with prodromal symptoms of high fevers, dizziness, and body aches. This prodromal phase is associated with higher disease severity, which may last 1 to 3 days [41,42]. The onset of symptoms usually occurs 24 to 72 h after ingestion of the bacteria. Common symptoms that occur during the acute phase of illness include abdominal cramping and multiple episodes of diarrhea, sometimes greater than 10 episodes during a 24 h period [42]. Bloody and mucous-like stools are common and result from the invasion of the intestinal epithelium by the bacteria [42]. Of note, the patient’s abdominal pain may mimic appendicitis due to acute ileocolitis [42]. Treatment revolves around hydration and electrolyte repletion. Depending on the severity of illness, patients can either be provided with oral or intravenous hydration [41]. Antibiotic therapy is only considered for patients who are immunocompromised or individuals with advanced age. Additionally, those with fever, abdominal pain, and bloody stools can also be considered for antibiotics [42]. When it comes to antibiotic therapy, Azithromycin remains the treatment of choice, followed by fluoroquinolones such as Levofloxacin and Ciprofloxacin. It is important to note that over time, resistant strains of *Campylobacter* have emerged, and those isolates have been noted to have higher resistance to fluoroquinolones compared to Azithromycin [41,42]. Antimicrobial therapy should be 3 days or longer, depending on host risk factors [42]. For individuals who are immunosuppressed (i.e., HIV), antibiotics are warranted for 7–14 days.

Complications

There are numerous complications associated with *Campylobacter* infection:

- **Guillain-Barre syndrome (GBS):** *C. jejuni* is responsible for 30% of all clinically significant GBS infections. This disorder is known to cause symmetrical ascending flaccid paralysis due to molecular mimicry, creating auto-antibodies that react with peripheral nerves [42].
- **Reactive arthritis:** commonly described in young adults. This has been described in approximately 2 to 5% of patients. These symptoms usually begin 4 weeks after infection, and the condition predominantly affects the knees and ankles [44,45].
- **Irritable bowel syndrome:** another post-infectious complication that has been described in 9 to 13% of infected patients [44,45].

Prevention continues to play a key role in avoiding the spread of viral and bacterial gastroenteritis alike. When it comes to mitigating *Campylobacter* spread, individuals should be careful to properly sanitize utensils, cutting boards, and other items that are used to cook raw poultry. Additionally, avoidance of unpasteurized dairy products, such as milk and cheese, is also an important preventative measure [41,46]. The CDC recommends washing any items that come into contact with raw poultry to avoid cross-contamination [41].

#### 3.5.3. *Shigella*

##### Epidemiology

*Shigella* remains one of the top three leading causes of bacterial gastroenteritis worldwide and in the United States [32,47]. It remains the third most common cause of gastroenteritis in the United States, and there are approximately 500,000 cases of *shigellosis* annually [47].

The four major strains of *Shigella* include *S. sonnei*, *S. flexneri*, *S. dysenteriae*, and *S. boydii.* Of those four strains, *S. Sonnei* is the most common species found in the United States and other developed nations [45,48]. Infections caused by *S. dysenteriae and S. boydii* are rare in the United States, but they remain common causes of infections worldwide. Of note, *S. boydii* is mostly restricted to the Indian sub-continent, while *S. dysenteriae* is common in sub-Saharan Africa and South Asia [49].

##### Transmission

*Shigella* is transmitted via the fecal–oral route, through direct person-to-person contact or indirectly through contaminated food, water, or fomites [49]. Outbreaks of shigellosis tend to occur in places where sanitation and hygiene practices are inadequate, which commonly include schools, daycare centers, private residences, and restaurants. Additionally, most outbreaks of shigellosis in the United States are seen in travelers returning from endemic regions, and recent evidence has also revealed increasing transmission rates via sexual contact, especially between men who have sex with men [49,50]. Although the bacteria affects all types of populations, it is important to note that very young children, the elderly, and immunocompromised individuals are at significantly increased risk for acquiring shigellosis [50].

Once acquired, only a small inoculum, as few as 10 to 100 organisms, is required to cause an infection. *Shigella* is not as prone to being neutralized by gastric acid, and thus, once the bacteria leaves the stomach, the organism multiplies in the small intestine and enters the colon, where it secretes enterotoxins, leading to invasion of the colonic epithelium [51]. Shigella strains produce three distinct enterotoxins, ShET2 (produced by all four species), ShET1 (produced by *S. flexneri*), and Shiga toxin (produced by *S. dysenteriae*, *S. Sonnei*, *S. flexneri*). These strains then allow the organism to induce intestinal secretion of solutes and water [52]. The enterocyte injury caused by the enterotoxins leads to the creation of ulcers in the colon, which then leads to hemorrhagic or bloody diarrhea symptoms in the disease course [50].

##### Diagnosis, Clinical Presentation, and Treatment

The incubation period is usually 1–3 days, and patients usually present with constitutional symptoms such as fever, anorexia, and malaise. Initially, there is watery diarrhea that can subsequently contain blood and mucus. Additionally, tenesmus and abdominal pain remain common complaints [52]. Diagnosis is confirmed with stool culture, and a rapid-polymerase chain reaction (PCR) test is also becoming increasingly available in the United States [49,52].

Complications

Complications of Shigella are intestinal and extraintestinal:

- **Bacteremia:** Noted in children < 5 and adults > 65 years of age. Young, malnourished children are at higher risk [50,52].
- **Toxic Megacolon:** Occurs primarily in the setting of *S. dysenteriae* 1 infection. The pathophysiology remains uncertain, but it is noted to occur in the setting of pancolitis [50,52].
- **Proctitis or rectal prolapse:** Usually seen in infants and young children, likely due to severe inflammation of the rectum and distal colon [50].
- **Colonic perforation:** Extremely rare complication that mostly affects infants or severely malnourished patients. Associated with *S. dysenteriae 1* or *S. flexneri* [50].
- **Seizures:** Most common complication; almost exclusively seen in children < 15 years of age. Seizures are seen with all serotypes of *Shigella I* [50,52].
- **Other manifestations:** Hyponatremia, reactive arthritis, vulvovaginitis with or without diarrhea, keratitis, and acute myocarditis have also been observed as extra-intestinal manifestations of shigellosis [50,52].

The treatment for shigellosis in immunocompetent individuals is usually supportive care, as the infection resolves within 5 to 7 days. Of note, anti-motility agents are not recommended for these infections as they can worsen the condition and put patients at risk for toxic megacolon [53]. Antibiotics are recommended in patients who are immunocompromised, have severe disease such as bacteremia, intestinal or extraintestinal complications, and need hospitalization [52]. Additionally, giving antibiotics will shorten the duration of fever and diarrhea symptoms by 2 days and may also shorten pathogen shedding in the stool. This will indirectly benefit public health by reducing person-to-person spread [52]. The choice of antibiotic therapy should depend on certain criteria, which involve considering recent travel history, especially if the patient has traveled to Asia or Africa, sexual contacts, previous antimicrobial use, homelessness, and immunosuppression. For patients who have no clear risk factors, treating with a fluoroquinolone is the treatment of choice. Alternatively, Azithromycin and Trimethoprim-Sulfamethoxazole are also acceptable treatment options [49,52]. Treating with carbapenems (such as Ertapenem, Imipenem, or Meropenem) can be considered in immune-compromised individuals, HIV patients, and men who have sex with men. It is important to note that these populations are at higher risk for severe infections and susceptible to acquiring resistant forms of shigellosis [49,52].

Disease prevention remains very important when trying to prevent spread. In order to prevent the spread of shigellosis, proper handwashing, staying home from school, food service, healthcare, or childcare jobs will help curb the spread. Additionally, avoiding any sexual contact with symptomatic patients, abstaining from using swimming pools, hot tubs, oceans, lakes, and rivers is also recommended [52].

#### 3.5.4. *Escherichia coli*

*Escherichia coli* (*E. coli*) is a Gram-negative bacillus that is implicated in causing multiple systemic infections in humans. It continues to be a significant burden on patients and healthcare systems alike. *E. coli* is a causative organism in intestinal infections, such as travelers’ diarrhea and dysentery, in addition to extra-intestinal manifestations leading to uncomplicated cystitis, pneumonia, bacteremia, and spontaneous bacterial peritonitis [53]. For the purpose of this review, we will discuss the epidemiology and clinical characteristics *of E. coli*-induced gastroenteritis. It is important to note that intestinal illnesses caused by *E. coli* are due to one of five subtypes [53]. Each subtype differs by epidemiology, as some strains are more abundant in resource-rich settings, whereas others are commonly found in resource-limited settings.

Of note, there are six pathotypes of *E. coli* that cause diarrhea. The specific serotypes of *E. coli* are determined by the surface antigens (O and H), and specific serotypes tend to cluster with certain pathotypes. The O antigen is determined by a repeating polysaccharide chain present on the lipopolysaccharide outer membrane (LPS), and the flagellum determines the H antigen [54,55].

Table 2 discusses multiple strains of *E. coli* and disease epidemiology.

Note: Specific global incidence and mortality data for EPEC, EIEC, and EAEC are limited or not well-defined in the available literature.

As noted above, most cases of *E. coli* diarrhea can be treated conservatively, and only the most severe cases require antibiotic therapy. Treatment for more severe forms of gastroenteritis should always take into account certain host factors. Consideration for initiating antibiotic therapy early should be given to patients who are advanced in age (>70) and immunocompromised. Of note, not all forms of bacterial *E. coli* infections require antibiotic therapy. The *E. coli 0157:H7* strain produces Shiga toxin, and evidence exists that treating this strain with antibiotics leads to antibiotic-induced lysis of organisms, which increases the likelihood of HUS. Thus, when patients are diagnosed with this form of *E. coli*, those patients should be treated conservatively with adequate hydration and supportive care [60,61].

As mentioned in all other forms of gastroenteritis, prevention remains a key factor in preventing disease spread. Refer to Table 2 for further details.

## 4. Discussion

Gastroenteritis remains a significant public health concern worldwide, with viral, bacterial, and parasitic etiologies contributing to morbidity and mortality. This review highlights the most common pathogens involved in acute gastroenteritis, emphasizing their epidemiology, clinical presentation, diagnostic criteria, treatment modalities, and preventative strategies.

Viral gastroenteritis, particularly due to norovirus and rotavirus, continues to be a major cause of diarrheal illnesses globally. While rotavirus vaccination has significantly reduced disease incidence, norovirus remains a persistent challenge due to its high transmissibility and genetic variability. Emerging research into norovirus vaccines and antiviral therapies shows promise but requires further development. Similarly, the increasing recognition of sapovirus and astrovirus as significant contributors to viral gastroenteritis suggests that expanded diagnostic capabilities and targeted preventive measures are needed.

Bacterial gastroenteritis, predominantly caused by non-typhoidal *Salmonella*, *Campylobacter*, *Shigella*, and *Escherichia coli*, presents with a range of clinical severities. While many infections are self-limited, antibiotic resistance remains a growing concern, particularly in Salmonella and Shigella species. The emergence of fluoroquinolone-resistant Campylobacter and multidrug-resistant *Shigella* underscores the need for improved antimicrobial stewardship.

Preventative measures such as improved sanitation, hand hygiene, food safety regulations, and vaccination programs have played a crucial role in reducing the burden of gastroenteritis. However, gaps remain in addressing outbreaks in healthcare and long-term care facilities, particularly among vulnerable populations such as the elderly and immunocompromised individuals. Future research should focus on novel vaccine candidates, rapid diagnostic tools, and targeted therapeutic interventions to mitigate the global impact of gastroenteritis.

## 5. Conclusions

Acute gastroenteritis remains a major cause of morbidity worldwide, with viral, bacterial, and parasitic pathogens contributing to the disease burden. While vaccination has proven effective in reducing rotavirus-associated infections, norovirus continues to pose a significant challenge. Bacterial gastroenteritis, particularly with increasing antibiotic resistance, necessitates ongoing surveillance and antimicrobial stewardship.

Enhanced diagnostic methods, improved sanitation efforts, and continued research into novel therapeutics and vaccines are essential to curbing the impact of gastroenteritis. Public health initiatives focusing on hygiene practices, outbreak containment, and global vaccine distribution will be critical in managing the disease burden. Moving forward, a multidisciplinary approach integrating epidemiological surveillance, clinical research, and public health interventions will be vital in reducing the incidence and severity of gastroenteritis worldwide.

In addition, greater emphasis should be placed on global health initiatives to improve water sanitation, food safety, and access to medical care, particularly in low-income regions where gastroenteritis-related mortality remains high. Investment in education and awareness campaigns can further empower individuals and communities to adopt preventative health measures. By fostering collaborations between governmental agencies, research institutions, and healthcare providers, a comprehensive strategy can be implemented to minimize the burden of gastroenteritis and improve patient outcomes across diverse populations.

## Figures and Tables

**Table 1:** Table summarizing the viruses discussed, including their incidence, seasonality, treatment, and prevention.

Virus	Incidence	Seasonality	Populations Affected	Treatment	Prevention
**Norovirus**	Most common cause of gastroenteritis worldwide; responsible for 90% of diarrheal illnesses globally	More common in winter months	Children < 5 years, elderly > 85 years, residents of long-term care facilities, childcare centers, cruise ship passengers	Supportive care with fluid repletion, anti-emetics, and anti-motility agents	Hand hygiene, contact precautions in healthcare settings, outbreak control measures
**Rotavirus**	Previously leading cause of pediatric viral gastroenteritis; incidence reduced significantly after vaccination	Peaks in winter months	Infants and young children (ages 4–24 months), especially in daycare settings; adults usually asymptomatic or have mild disease	Supportive care with oral rehydration, anti-emetics	Vaccination (Rotateq, Rotarix), hand hygiene, sanitation
**Astrovirus**	Causes 2–9% of viral gastroenteritis cases, mainly in children under 2 years	Peaks in winter months	Young children (<2 years), outbreaks in schools, daycare centers, hospitals, and nursing homes	Supportive care with oral rehydration	Hand hygiene, sanitation, cleaning contaminated surfaces
**Sapovirus**	Prevalence varies (1–17% of diarrheal episodes worldwide); mostly affects young children and older adults	Occurs year-round	Children < 5 years, older adults > 60 years, those with exposure to contaminated water or food (e.g., shellfish)	Supportive care with oral rehydration, anti-emetics	Hand hygiene, proper disposal of fecal materials, cleaning contaminated surfaces
**Adenovirus**	Causes 2–6% of viral gastroenteritis cases; mainly affects infants and young children	Occurs year-round	Infants, young children, school-age children, young adults in close quarters (e.g., dormitories, military)	Supportive care with fluid hydration, fever management	Hand hygiene, contact precautions, sanitation

**Table 2:** Summary of various *E. coli*.

Strain	Key Virulence Factors	Clinical Features	Transmission	Diagnosis	Treatment	Estimated Annual Cases	Estimated Annual Deaths
**Enterotoxigenic *E. coli* (ETEC)**	Heat-labile (LT) and heat-stable (ST) toxins	Traveler’s diarrhea: watery diarrhea, nausea, abdominal cramps	Contaminated food and water	Stool PCR, ELISA for toxins	Supportive care, rehydration; antibiotics (e.g., azithromycin) for severe cases	Approximately 220 million globally, with 40,000 in the USA	Over 50,000 globally, primarily in children under 5 years old
**Enteropathogenic *E. coli* (EPEC)**	Adherence factors (bundle-forming pili)	Infantile diarrhea: watery diarrhea, vomiting	Contaminated food, person-to-person	Stool PCR, microscopy for characteristic adherence patterns	Supportive care, rehydration	Data are not readily available, as many labs do not test for this.	Data not specified in available literature
**Enterohemorrhagic *E. coli* (EHEC)**	Shiga-like toxin (Stx1, Stx2)	Hemorrhagic colitis: bloody diarrhea, abdominal pain; may cause HUS	Contaminated beef, produce, water	Stool PCR for *stx* genes, culture on sorbitol-MacConkey agar	Supportive care; avoid antibiotics (risk of HUS)	STEC O157: ~97,000 in the USA; STEC non-O157: ~169,000 in the USA	STEC O157: ~90 in the USA (2024)
**Enteroinvasive *E. coli* (EIEC)**	Invasion plasmid antigens (Ipa proteins)	Dysentery-like illness: fever, bloody/mucoid diarrhea	Contaminated food and water	Stool culture, PCR	Supportive care; antibiotics in severe cases (e.g., fluoroquinolones)	Data are not readily available, as many labs do not test for this.	Data not specified in available literature
**Enteroaggregative *E. coli* (EAEC)**	Aggregative adherence fimbriae (AAF)	Persistent watery diarrhea, often in children and immunocompromised patients	Contaminated food and water	Stool PCR, characteristic “stacked brick” adherence on microscopy	Supportive care; antibiotics (e.g., rifaximin) for prolonged symptoms	Data are not readily available, as many labs do not test for this.	Data not specified in available literature

Please refer to the index of references below [54,55,56,57,58,59].
